# Supplementary material for: A bacterial TIR-based immune system senses viral capsids to initiate defense
Source: bioRxiv. 2024 Jul 29:2024.07.29.605636. Preprint. [Version 1] doi: 10.1101/2024.07.29.605636 (PMC11312562; doi:10.1101/2024.07.29.605636)
Supplement: 1 — Figure S1. Characterization of Sau-Thoeris defense. (A) Tenfold serial dilutions of Φ80α-vir on lawns of S. aureus RN4220 carrying either an incomplete (thsB1/B2) or full (thsA/B1/B2) Thoeris operon in a chromosomal location. (B) Growth of S. aureus RN4220 harboring plasmids carrying either an incomplete (thsB1/B2) or full (thsA/B1/B2) Thoeris operon, determined as the OD600 of the cultures after infection with Φ80α-vir at MOI 0, 0.1 or 10. Mean of +/− S.D. of three biological replicates is reported. Figure S2. Major head protein is required for Sau-Thoeris activation. (A) Schematic of ΦNM1 genome, with expansion of the operon for packaging the genome and assembly of phage particles. PE and PL are promoters responsible for the expression of early and late viral genes, respectively. The green asterisk indicates the nonsense mutation prevents ΦNM1 lysogeny, converting it into ΦNM1γ6, a purely lytic phage. Regions of the genome involved in different stages of the viral lytic cycle are indicated. (B) Genes of the ΦNM1 genome required for capsid biogenesis. (C) Tenfold serial dilutions of Φ80a-vir phage carrying wild-type or V273A mhp alleles on lawns of S. aureus RN4220 harboring plasmids carrying either an incomplete (thsB1/B2) or full (thsA/B1/B2) Thoeris operon. (D) Tenfold serial dilutions of lysates obtained after MMC induction of different ΦNM1 lysogens carrying deletions on genes involved in packaging the genome and assembly of phage particles on lawns of S. aureus RN4220 harboring an empty vector control, or plasmids expressing the genes deleted in the prophages. Figure S3. ThsB1-ThsB2-Mhp copurification assays. (A) Tenfold serial dilutions of Φ80a-vir phage on lawns of S. aureus RN4220 harboring plasmids carrying either untagged or hexahystidyl- or FLAG-tagged versions of ThsB1 or ThsB2, along with untagged ThsA. (B) LC-MS/MS identification of ~35 kDa protein isolated with ThsB1 and ThsB2 from infected S. aureus RN4220 cells shown in Figure 3B. Gel slices (n=3) [file NIHPP2024.07.29.605636V1-supplement-1.pdf]

bioRxiv preprint doi: https://doi.org/10.1101/2024.07.29.605636; this version posted July 29, 2024. The copyright holder for this preprint (which was not certified by peer review) is the author/funder, who has granted bioRxiv a license to display the preprint in perpetuity. It is made available under aCC-BY 4.0 International license.

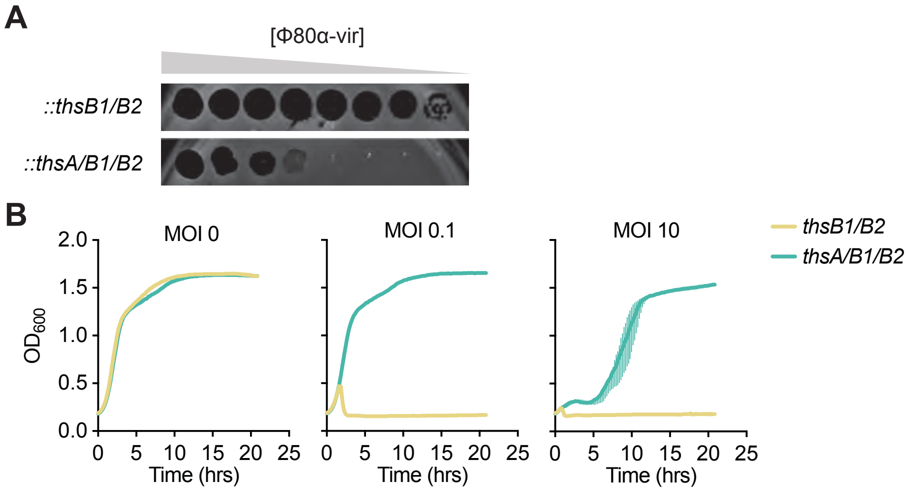

Figure S1. Roberts, Fishman *et al*.

bioRxiv preprint doi: https://doi.org/10.1101/2024.07.29.605636; this version posted July 29, 2024. The copyright holder for this preprint (which was not certified by peer review) is the author/funder, who has granted bioRxiv a license to display the preprint in perpetuity. It is made available under aCC-BY 4.0 International license.

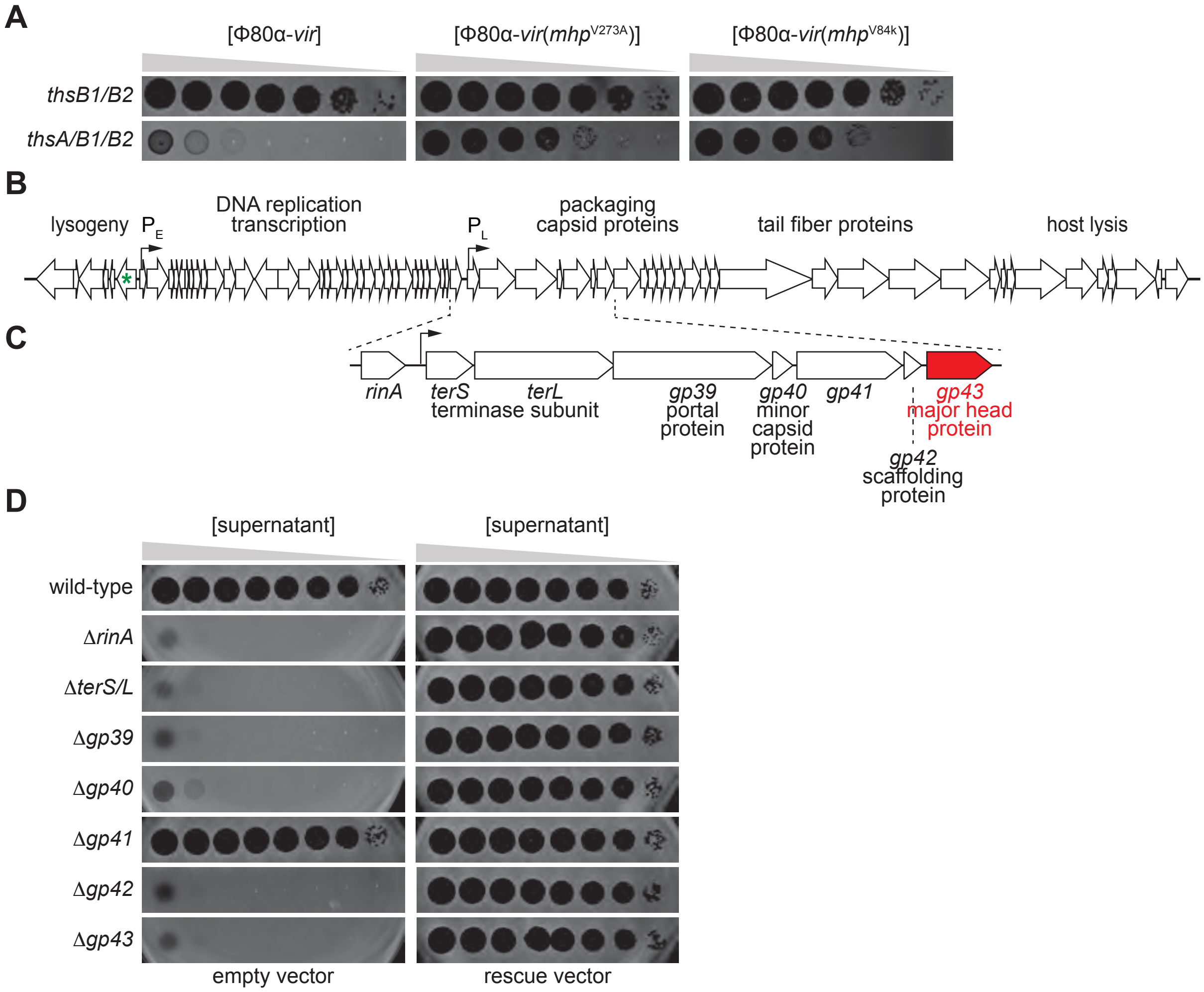

Figure S2. Roberts, Fishman *et al*.

bioRxiv preprint doi: https://doi.org/10.1101/2024.07.29.605636; this version posted July 29, 2024. The copyright holder for this preprint (which was not certified by peer review) is the author/funder, who has granted bioRxiv a license to display the preprint in perpetuity. It is made available under aCC-BY 4.0 International license.

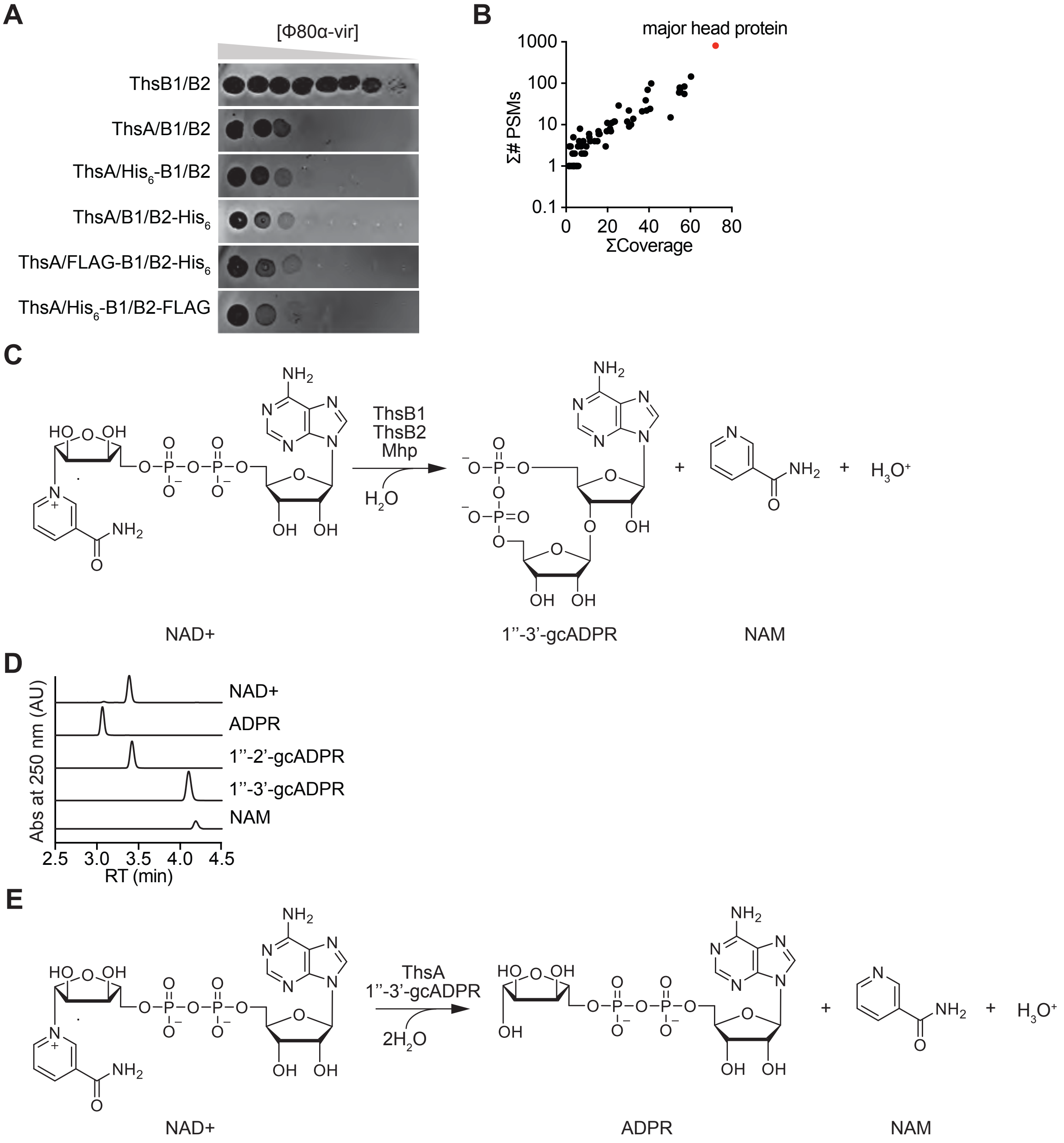

Figure S3. Roberts, Fishman *et al*.

bioRxiv preprint doi: https://doi.org/10.1101/2024.07.29.605636; this version posted July 29, 2024. The copyright holder for this preprint (which was not certified by peer review) is the author/funder, who has granted bioRxiv a license to display the preprint in perpetuity. It is made available under aCC-BY 4.0 International license.

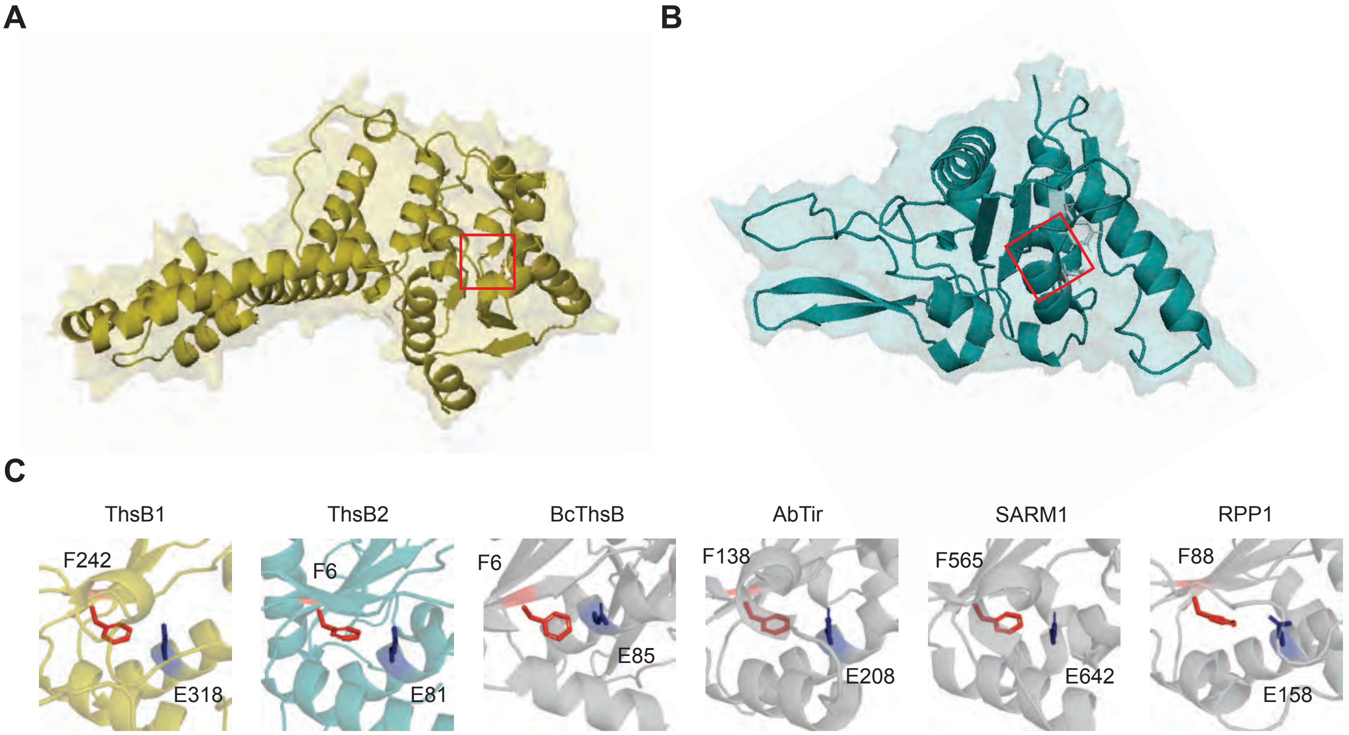

Figure S4. Roberts, Fishman *et al*.

bioRxiv preprint doi: https://doi.org/10.1101/2024.07.29.605636; this version posted July 29, 2024. The copyright holder for this preprint (which was not certified by peer review) is the author/funder, who has granted bioRxiv a license to display the preprint in perpetuity. It is made available under aCC-BY 4.0 International license.

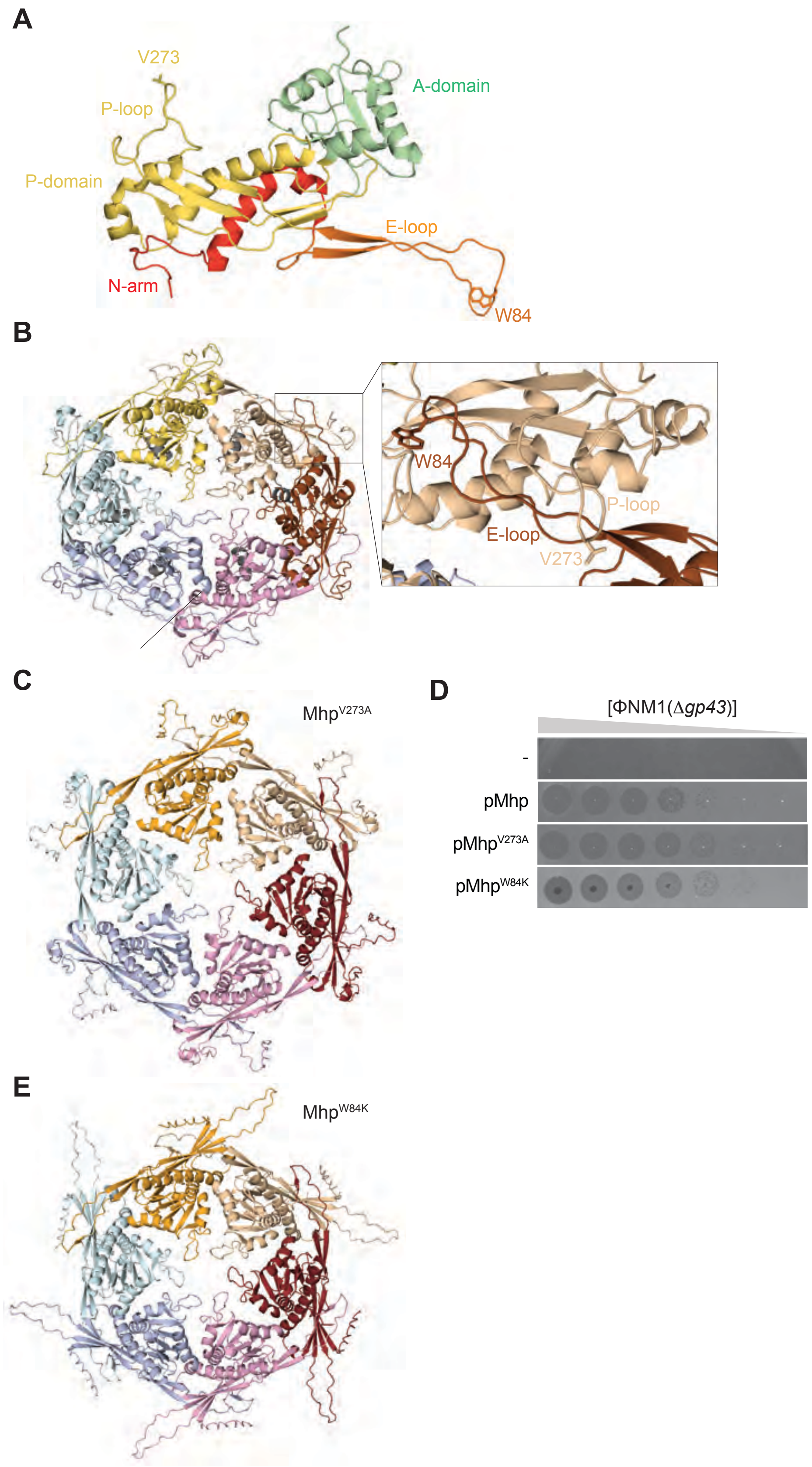

Figure S5. Roberts, Fishman *et al*.

bioRxiv preprint doi: https://doi.org/10.1101/2024.07.29.605636; this version posted July 29, 2024. The copyright holder for this preprint (which was not certified by peer review) is the author/funder, who has granted bioRxiv a license to display the preprint in perpetuity. It is made available under aCC-BY 4.0 International license.

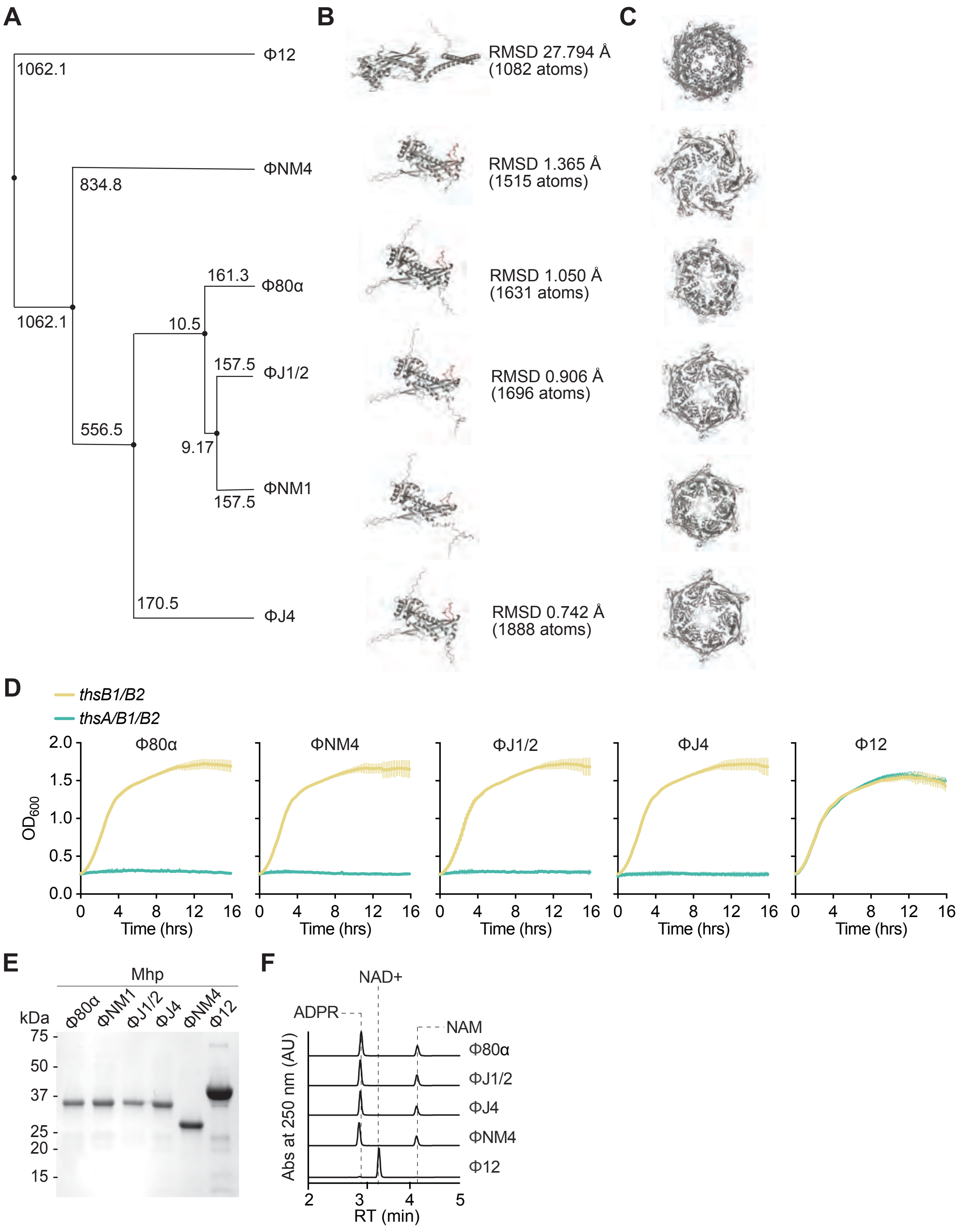

Figure S6. Roberts, Fishman *et al*.

bioRxiv preprint doi: https://doi.org/10.1101/2024.07.29.605636; this version posted July 29, 2024. The copyright holder for this preprint (which was not certified by peer review) is the author/funder, who has granted bioRxiv a license to display the preprint in perpetuity. It is made available under aCC-BY 4.0 International license.

SUPPLEMENTARY METHODS TABLES.

Supplementary Methods Table 1. Bacterial strains used in this study.

**Species**	**Strain**	**Genotype**	**Origin**
S. aureus	RN4220	Wild type	Kreiswerth et al., Nature (1983)
S. aureus	RN4220	::ΦNM1	Goldberg et al., Nature (2014)
S. aureus	RN4220	::ΦNM1ΔDnaC	Cre-loxP Recombineering (see methods)
S. aureus	RN4220	::ΦNM1ΔRinA	Cre-loxP Recombineering (see methods)
S. aureus	RN4220	::ΦNM1ΔTerS	Cre-loxP Recombineering (see methods)
S. aureus	RN4220	::ΦNM1ΔTerL	Cre-loxP Recombineering (see methods)
S. aureus	RN4220	::ΦNM1ΔPortal	Cre-loxP Recombineering (see methods)
S. aureus	RN4220	::ΦNM1ΔGp40	Cre-loxP Recombineering (see methods)
S. aureus	RN4220	::ΦNM1ΔGp41	Cre-loxP Recombineering (see methods)
S. aureus	RN4220	::ΦNM1ΔGp42	Cre-loxP Recombineering (see methods)
S. aureus	RN4220	::ΦNM1ΔMajor Head	Cre-loxP Recombineering (see methods)
S. aureus	RN4220	::Φ80α ΔMajor Head	Cre-loxP Recombineering (see methods)
S. aureus	RN4220	::Sau-Thoeris-ermR	Chromosomal integration (see methods)
S. aureus	RN4220	::Sau-ThsB1-B2-ermR	Chromosomal integration (see methods)

bioRxiv preprint doi: https://doi.org/10.1101/2024.07.29.605636; this version posted July 29, 2024. The copyright holder for this preprint (which was not certified by peer review) is the author/funder, who has granted bioRxiv a license to display the preprint in perpetuity. It is made available under aCC-BY 4.0 International license.

Supplementary Methods Table 2. Phages used in this study.

Phage	Host	Genotype	Origin
Φ80α-vir	*S. aureus*	Wild type	Banh and Roberts et al., Nature (2023)
Φ80α-vir^{GFP}	*S. aureus*	Wild type	Banh and Roberts et al., Nature (2023)
Φ80α-vir(gp47^{V273A})	*S. aureus*	Major head (gp47) V273>A (T818>C)	This study; isolated from screen for Sau-Thoeris escapers
Φ80α-vir(gp47^{W84K})	*S. aureus*	Major head (gp47) W84>K (TGG249-251>AAA)	This study; engineered by recombination using pCR186 and selected for using pCR187
ΦJ1	*S. aureus*	Wild type	Banh and Roberts et al., Nature (2023)
ΦJ2	*S. aureus*	Wild type	Banh and Roberts et al., Nature (2023)
ΦJ4	*S. aureus*	Wild type	Banh and Roberts et al., Nature (2023)
ΦNM1γ6	*S. aureus*	Wild type	Goldberg et al., Nature (2014)
ΦNM4γ4	*S. aureus*	Wild type	Heler et al., Nature (2015)
Φ12γ3	*S. aureus*	Wild type	Modell et al., Nature (2017)

bioRxiv preprint doi: https://doi.org/10.1101/2024.07.29.605636; this version posted July 29, 2024. The copyright holder for this preprint (which was not certified by peer review) is the author/funder, who has granted bioRxiv a license to display the preprint in perpetuity. It is made available under aCC-BY 4.0 International license.

Supplementary Methods Table 3. Plasmids used in this study.

Plasmid	Description	Source	Construction Notes
pPM300	Recombineering genes from phage ϕ11 under the control of an IPTG-inducible promoter and cre-recombinase under the control of an aTc-inducible promoter	Banh et al., 2023	
pDVB223	Thoeris operon from S. aureus 08BA02176, IPTG-inducible pE194-based vector for recombinant protein expression	This study	Gibson assembly: oDVB796+oDVB797 (S. aureus 08BA02176 template) oDVB_ + oDVB_ (pE194 template)
pCF8	ThsA+ThsB1, IPTG-inducible pE194-based vector for recombinant protein expression	This study	Gibson assembly: oCF.36+oCF.37 (pDVB223 template)
pCF9	ThsA+ThsB2, IPTG-inducible pE194-based vector for recombinant protein expression	This study	Gibson assembly: oCF.38+oCF.39 (pDVB223 template)
pCF10	ThsA, IPTG-inducible pE194-based vector for recombinant protein expression	This study	Gibson assembly: oCF.40+oCF.41 (pDVB223 template)
pCF11	ThsB1+ThsB2 IPTG-inducible pE194-based vector for recombinant protein expression	This study	Gibson assembly: oCF.42+oCF.43 (pDVB223 template)
pCF34	His6-ThsA+ThsB1+ThsB2, IPTG-inducible pE194-based vector for recombinant protein expression and purification of ThsA	This study	Gibson assembly: oCF.70+oCF.91 oCF.86+oCF.90 (pDVB223 template)
pCF35	ThsA+His6-ThsB1+ThsB2, IPTG-inducible pE194-based vector for recombinant protein expression and purification of ThsB1	This study	Gibson assembly: oCF.72+oCF.91 oCF.73+oCF.90 (pDVB223 template)
pCF41	gp43 (major head) from ΦNM1γ6 with escape mutation (V273A), IPTG-	This study	Gibson assembly: oCF.84+oCF.85 (pCR176 template)

bioRxiv preprint doi: https://doi.org/10.1101/2024.07.29.605636; this version posted July 29, 2024. The copyright holder for this preprint (which was not certified by peer review) is the author/funder, who has granted bioRxiv a license to display the preprint in perpetuity. It is made available under aCC-BY 4.0 International license.

	inducible pC194-based vector for recombinant protein expression		
pCF45	N315 hypothetical protein from Φ12γ3, IPTG-inducible pC194-based vector for recombinant protein expression	This study	Gibson assembly: oCF.98+oCF.99 (Φ12γ3 template) oCF.97+oCF100 (pC194 template)
pCF46	His6-N315 hypothetical protein from Φ12γ3, IPTG-inducible pC194-based vector for recombinant protein expression	This study	Gibson assembly: oCF.86+oCF.101 (pCF.45 template)
pCF47	ThsB1(F242A)+ThsB2 IPTG-inducible pE194-based vector for recombinant protein expression	This study	Gibson Assembly: oCF.102+oCF.103 (pCF.11 template)
pCF48	ThsB1(E318Q)+ThsB2 IPTG-inducible pE194-based vector for recombinant protein expression	This study	Gibson Assembly: oCF.104+oCF.105 (pCF.11 template)
pCF49	ThsB1+ThsB2(F6A) IPTG-inducible pE194-based vector for recombinant protein expression	This study	Gibson Assembly: oCF.106+oCF.107 (pCF.11 template)
pCF50	ThsB1+ThsB2(E81Q) IPTG-inducible pE194-based vector for recombinant protein expression	This study	Gibson Assembly: oCF.108+oCF.109 (pCF.11 template)
pCF51	ThsA+ThsB1+ThsB2-His6, IPTG-inducible pE194-based vector for recombinant protein expression and purification of ThsB2	This study	Gibson Assembly: oCF.110+oCF.111 (pDVB223 template)
pCF52	His6-ThsB1+ThsB2-3xFlag, IPTG-inducible pE194-based vector for recombinant protein expression and purification of ThsB1 and ThsB2	This study	Gibson Assembly: oCF.112 + oCF.114 oCF.72 + oCF.113 oCF.86 + oCF.115 (pCF.11 template)
pCF53	3xFlag-ThsB1+ThsB2-His6, IPTG-inducible pE194-based vector for recombinant protein expression and	This study	oCF.112 + oCF.117 oCF.78 + oCF.113 oCF.87 + oCF.116 (pCF.11 template)

bioRxiv preprint doi: https://doi.org/10.1101/2024.07.29.605636; this version posted July 29, 2024. The copyright holder for this preprint (which was not certified by peer review) is the author/funder, who has granted bioRxiv a license to display the preprint in perpetuity. It is made available under aCC-BY 4.0 International license.

	purification of ThsB1 and ThsB2		
pCF55	ThsA+ThsB1(F242A)+ThsB2 IPTG-inducible pE194-based vector for recombinant protein expression	This study	Gibson Assembly: oCF.102+oCF.103 (pDVB223 template)
pCF56	ThsA+ThsB1(E318Q)+ThsB2 IPTG-inducible pE194-based vector for recombinant protein expression	This study	Gibson Assembly: oCF.104+oCF.105 (pDVB223 template)
pCF57	ThsA+ThsB1+ThsB2(F6A) IPTG-inducible pE194-based vector for recombinant protein expression	This study	Gibson Assembly: oCF.106+oCF.107 (pDVB223 template)
pCF58	ThsA+ThsB1+ThsB2(E81Q) IPTG-inducible pE194-based vector for recombinant protein expression	This study	Gibson Assembly: oCF.108+oCF.109 (pDVB223 template)
pCF60	ThsB1+ThsB2 IPTG-inducible pE194-based vector for recombinant protein expression	This study	Gibson assembly: oCF.110+oCF.111 (pCF.11 template)
pCF64	Gp43(W84K) (Major Head) of ΦNM1γ6, IPTG-inducible pC194-based vector for recombinant protein expression	This study	Gibson assembly: oCF.126+oCF.127 (pCR176 template)
pCF69	His-ThsB1 IPTG-inducible pE194-based vector for recombinant protein expression	This study	Gibson Assembly: oCF.36+oCF.37 (pCF.52 template)
pCF70	ThsB2-His IPTG-inducible pE194-based vector for recombinant protein expression	This study	Gibson Assembly: oCF.136+oCF.137 (pCF.53 template)
pCF80	ThsA+His6-ThsB1+ThsB2-3xFLAG, IPTG-inducible pE194-based vector for recombinant protein expression	This study	Gibson Assembly oCF.114+oCF.115 (template pCF.35)
pCF81	ThsA+3xFLAG-ThsB1+ThsB2-His6, IPTG-inducible pE194-based vector for recombinant protein expression	This study	Gibson Assembly oCF.78+oCF.160 (template pCF.51)

bioRxiv preprint doi: https://doi.org/10.1101/2024.07.29.605636; this version posted July 29, 2024. The copyright holder for this preprint (which was not certified by peer review) is the author/funder, who has granted bioRxiv a license to display the preprint in perpetuity. It is made available under aCC-BY 4.0 International license.

pCR173	terS-Gp43 (full packaging and structure operon) of ΦNM1γ6, IPTG-inducible pC194-based vector for recombinant protein expression	This study	Gibson Assembly: oCR499+oCR500 (ΦNM1γ6 template) oCR497+oCR498 (pC194 template)
pCR174	Portal-Gp43 of ΦNM1γ6, IPTG-inducible pC194-based vector for recombinant protein expression	This study	Gibson Assembly: oCR501+oCR500 (ΦNM1γ6 template) oCR497+oCR498 (pC194 template)
pCR175	Gp40-Gp43 of ΦNM1γ6, IPTG-inducible pC194-based vector for recombinant protein expression	This study	Gibson Assembly: oCR502+oCR500 (ΦNM1γ6 template) oCR497+oCR498 (pC194 template)
pCR176	Gp43 (Major Head) of ΦNM1γ6, IPTG-inducible pC194-based vector for recombinant protein expression	This study	Gibson Assembly: oCR503+oCR500 (ΦNM1γ6 template) oCR497+oCR498 (pC194 template)
pCR177	terL (large terminase subunit) of ΦNM1γ6, IPTG-inducible pC194-based vector for recombinant protein expression	This study	Gibson Assembly: oCR504+oCR505 (ΦNM1γ6 template) oCR497+oCR498 (pC194 template)
pCR178	terS-terL of ΦNM1γ6, IPTG-inducible pC194-based vector for recombinant protein expression	This study	Gibson Assembly: oCR499+oCR505 (ΦNM1γ6 template) oCR497+oCR498 (pC194 template)
pCR179	Major Head of ΦJ1/2, IPTG-inducible pC194-based vector for recombinant protein expression	This study	Gibson Assembly: oCR497+oCR498 (pCR176 template) oCR515+oCR516 (ΦJ1/2 template)
pCR180	Major Head of ΦJ4, IPTG-inducible pC194-based vector for recombinant protein expression	This study	Gibson Assembly: oCR497+oCR498 (pCR176 template) oCR517+oCR518 (ΦJ4 template)
pCR181	Major Head of Φ80α, IPTG-inducible pC194-based	This study	Gibson Assembly: oCR497+oCR498

bioRxiv preprint doi: https://doi.org/10.1101/2024.07.29.605636; this version posted July 29, 2024. The copyright holder for this preprint (which was not certified by peer review) is the author/funder, who has granted bioRxiv a license to display the preprint in perpetuity. It is made available under aCC-BY 4.0 International license.

	vector for recombinant protein expression		(pCR176 template) oCR519+oCR520 (Φ80α template)
pCR184	Major Head of Φ80α with V273A, IPTG-inducible pC194-based vector for recombinant protein expression		Gibson assembly: oCF.84+oCF.85 (pCR181 template)
pCR185	Major Head of Φ80α with W84K, IPTG-inducible pC194-based vector for recombinant protein expression		Gibson assembly: oCF.126+oCF.127 (pCR181 template)
pCR182	Major Head of ΦNM4, IPTG-inducible pC194-based vector for recombinant protein expression	This study	Gibson Assembly: oCR497+oCR498 (pCR176 template) oCR521+oCR522 (ΦNM4 template)
pCR183	Major Head of Φ12, IPTG-inducible pC194-based vector for recombinant protein expression	This study	Gibson Assembly: oCR497+oCR498 (pCR176 template) oCR523+oCR524 (Φ12 template)
pCR186	Recombination plasmid harboring *mhp*^{W84K} gene with 500-nt upstream and downstream homology arms corresponding to Φ80α *gp46* and *gp48*, respectively	This study	Gibson Assembly: oCR589+oCR590 (pCR176 template) oCR591+oCR592 (Φ80α gDNA template) oCR593+oCR594 (pCF64 template) oCR595+oCR596 (Φ80α gDNA template)
pCR187	*S. aureus* M06/0171 type II-A CRISPR-Cas system with programmed spacer targeting wild-type Φ80α-vir	This study	Ligation of BsaI-digested pDVB47 (Banh et al., 2023) and annealed oCR597/oCR598

bioRxiv preprint doi: https://doi.org/10.1101/2024.07.29.605636; this version posted July 29, 2024. The copyright holder for this preprint (which was not certified by peer review) is the author/funder, who has granted bioRxiv a license to display the preprint in perpetuity. It is made available under aCC-BY 4.0 International license.

	but not Φ80α-vir::*mhp*^{W84K} (mutation changes PAM)		

bioRxiv preprint doi: https://doi.org/10.1101/2024.07.29.605636; this version posted July 29, 2024. The copyright holder for this preprint (which was not certified by peer review) is the author/funder, who has granted bioRxiv a license to display the preprint in perpetuity. It is made available under aCC-BY 4.0 International license.

Supplementary Methods Table 4. Oligonucleotide primers used in this study.

Primer	Sequence
oCR24	ATCAATCAAACGTTAATCCGTCTTTAAGAGATGCAACAGTCAAAAACTACAAGCCATAACTTCGTATAGCATACATTATACGAAGTTATAGTGACATTAG
oCR25	AGCCTTGTACGTACTCTATTGCTGTTTGTTTAGCGTGTACTTGTTTTTCATTTTGTATAACTTCGTATAATGTATGCTATACGAAGTTATTATATTTATG
oCR26	ATACAAATATAGGCGGGGAGTTTGTACCGTCTAATACATCAAAAACAGAAATGGCAGTAACATAACTTCGTATAGCATACATTATACGAAGTTATAGTGACATTAG
oCR27	CGTTCAATCGCACTCTTAAACTCAAGAATTTTACCTCTTCGTATACTACAAAGATAATTAATAACTTCGTATAATGTATGCTATACGAAGTTATTATATTTATG
oCR59	ATCTGCTTCTATTGCTAGAGGAGAACCTCAAGAGGCTTACAGTAAGAAATATGACCATTTATAACTTCGTATAGCATACATTATACGAAGTTATAGTGACATTAG
oCR60	TGACGCTCTTCAAAAGTTGGTGTGATTGTGTAAGTAACCTCTTTTTCCACTTCATCGTTTATAACTTCGTATAATGTATGCTATACGAAGTTATTATATTTATG
oCR63	TTAAGAACTGGAAGAAGAAACCAGGTAAGCCGTTAGACGTACAACTTGATGAATTAGCTGATAACTTCGTATAGCATACATTATACGAAGTTATAGTGACATTAG
oCR64	TTTATCTCTTCTGATGACACTCCTACTTGATTCGCAATACTCAATCCAAACGCCAACATGTATAACTTCGTATAATGTATGCTATACGAAGTTATTATATTTATG
oCR95	AAATAATCATCCTCCTAAGTACAAGCTTAATTGTTATCCGCTCACAATTCCACACATTAT
oCR96	GAGTGATCGTTAAATTTATACTGCAATCGGATGCGATTATTGAATAAAAGATATGAGAGA
oCR482	GGTGTTGAAACGCGATACTTTTCTAATAATGATAGCGAACTATTGAAGAGTCACATGTTTTATTGGAGTGTTTATGCATCCCTTAACTTACTTATTAAAT
oCR483	GCTAATTGACAAGGTCTCATAAATGACTCAGCAAACGATTGCAATGTATTGATACGGTTATTCTGTTTATTAAAGCATTAAAACCCCATGAATTATTTT
oCR484	GCTAATTGACAAGGTCTCATAAATGACTCAGCAAACGATTGCAATGTATTGATACGGTTATTCTGTTTATTTATTTCTTCTACAGATATTATAATTCGTTGC
oCR485	ACGACAAAACTTAGAGTTGTTAGCAAATCGTAATCCAGCATATTACAAAATTTATGCGTTATAACTTCGTATAGCATACATTATACGAAGTTATAGTGACATTAG

bioRxiv preprint doi: https://doi.org/10.1101/2024.07.29.605636; this version posted July 29, 2024. The copyright holder for this preprint (which was not certified by peer review) is the author/funder, who has granted bioRxiv a license to display the preprint in perpetuity. It is made available under aCC-BY 4.0 International license.

oCR486	TTTATTAAACGTTTTTCATACTTAGGGAAAACCAATTTGTCTAGTGTAGCAAATTCACCTATAACTTCGTATAATGTATGCTATACGAAGTTATTATATTTATG
oCR487	GTTACTTATTAAAGGTAATTTAAATTTAGATCCCGTAGAAGTTAGAAAACAAAAGGAAGCATAACTTCGTATAGCATACATTATACGAAGTTATAGTGACATTAG
oCR488	CCTTCTGTTTCTCTACCTTCGCTATCAGCATAAACAGTCGGTTCTAAAAACAACACGTTAATAACTTCGTATAATGTATGCTATACGAAGTTATTATATTTATG
oCR489	AATAATTGCTAATGTAGTTATTAGAGGTCGACATCCTAATGAATATGTTAAAGATATGCGATAACTTCGTATAGCATACATTATACGAAGTTATAGTGACATTAG
oCR490	AATGATTTAATTGCTGCGGTCTTTTGTCGTGCTGTGCCTTCGAATTTATTTAAGTGCTTGATAACTTCGTATAATGTATGCTATACGAAGTTATTATATTTATG
oCR491	TAGAGACATAGCAAGAGAGTTAAAAGGTATACGTAAAGAGTTACAAAAGCGAAACGAAACATAACTTCGTATAGCATACATTATACGAAGTTATAGTGACATTAG
oCR492	TTTTCTTTATCGGCTAATACTGCCGACCTTACGCTGTCTAAGTTTGCATCAATAATAACTATAACTTCGTATAATGTATGCTATACGAAGTTATTATATTTATG
oCR493	TAAAGAAGAATTAAGTCGTCGTATGAAGCAGAAAGAAAAAGAGAAACAAGAAGCTGTTGAATAACTTCGTATAGCATACATTATACGAAGTTATAGTGACATTAG
oCR494	TGTTCGCGTTCATATTCAGCGATTTGATCTTTGTTCATTTTTGCTAATCGTTTAGCTTCAATAACTTCGTATAATGTATGCTATACGAAGTTATTATATTTATG
oCR495	AGGTGAAGGTCAAAAAATCGAAACATCTAAAGCTACATGGGTTAATGCTACTATGAGAGCATAACTTCGTATAGCATACATTATACGAAGTTATAGTGACATTAG
oCR496	TGTGAATAAGTGTAATTCAAAAATTCTTTTGTTACAGGTAAGATAACCCCTAATTTAAACATAACTTCGTATAATGTATGCTATACGAAGTTATTATATTTATG
oCR497	AAATAATCATCCTCCTAAGTACAAGCTTAA
oCR498	GACGTGGTTTAACCCGGGTAACTAGTAACT
oCR499	TTAAGCTTGTACTTAGGAGGATGATTATTTATGAACGAAAAACAAAAGAGATTCGC
oCR500	AGTTACTAGTTACCCGGGTTAAACCACGTCTTAAACTTCTCCTGGTACTGAATCTGT
oCR501	TTAAGCTTGTACTTAGGAGGATGATTATTTATGTTAAAAGTAAACGAATTTGAAACAGAT
oCR502	TTAAGCTTGTACTTAGGAGGATGATTATTTTTGCCTAACAAAAACACTCAAGAATATTG
oCR503	TTAAGCTTGTACTTAGGAGGATGATTATTTATGGAACAAACACAAAAATTAAAATTAAAT

bioRxiv preprint doi: https://doi.org/10.1101/2024.07.29.605636; this version posted July 29, 2024. The copyright holder for this preprint (which was not certified by peer review) is the author/funder, who has granted bioRxiv a license to display the preprint in perpetuity. It is made available under aCC-BY 4.0 International license.

oCR504	TTAAGCTTGTACTTAGGAGGATGATTATTTATGACGAAAGTTAAATTAAACTTTAACAAA
oCR505	AGTTACTAGTTACCCGGGTTAAACCACGTCCTATAATCCTAGAGATTTTATTGTGTCAAC
oCR515	TTAAGCTTGTACTTAGGAGGATGATTATTTATGGAACAAACACAAAAATTAAAATTAAAT
oCR516	AGTTACTAGTTACCCGGGTTAAACCACGTCTTAAACTTCTCCTGGTACTGAATC
oCR517	TTAAGCTTGTACTTAGGAGGATGATTATTTATGGAACAAACACAAAAATTAAAATTAAAT
oCR518	AGTTACTAGTTACCCGGGTTAAACCACGTCTTAAACTTCTCCTGGAACTGAAG
oCR519	TTAAGCTTGTACTTAGGAGGATGATTATTTATGGAACAAACACAAAAATTAAAATTAAAT
oCR520	AGTTACTAGTTACCCGGGTTAAACCACGTCTTAAACTTCTCCTGGAACTGAATCTG
oCR521	TTAAGCTTGTACTTAGGAGGATGATTATTTATGGCAACTCCAACATACAC
oCR522	AGTTACTAGTTACCCGGGTTAAACCACGTCCTATTCAGTTGGTTTAAGCGTTGC
oCR523	TTAAGCTTGTACTTAGGAGGATGATTATTTATGCGAAATTTTAAAAATGACAATGAATT
oCR524	AGTTACTAGTTACCCGGGTTAAACCACGTCTTAGCTGGGTAATGGACCTGTA
oCR589	ATTTTAAAAATATCCCACTTTATCCAATTTTCGT
oCR590	ATATATTTATGTTACAGTAATATTGACTTTTAAAAAAGG
oCR591	ACGAAAATTGGATAAAGTGGGATATTTTTAAAATCTGAAATAACCTTCACGCC
oCR592	ATTTAATTTTAATTTTTGTGTTTGTTCCATTTAAATGCCTCCGTTAATTTTTAATAATTC
oCR593	ATGGAACAAACACAAAAATTAAAATTAAAT
oCR594	TTAAACTTCTCCTGGTACTGAATCTGTTTT
oCR595	AAAACAGATTCAGTACCAGGAGAAGTTTAATAAACAATTAGGAGTGGTAACATGC
oCR596	CCTTTTTTAAAAGTCAATATTACTGTAACATAAATATATGTTGTAGCGTTTAACTGCAAC
oCR597	AAACACTTTTTGGGCTGATAAACCAGGTGCTTACG
oCR598	AAAACGTAAGCACCTGGTTTATCAGCCCAAAAAGT
oCF.36	GCTTTTTCAGATTAGGAGTGATCGTTAAATTTATACTGCAATCG
oCF.37	ATTTAACGATCACTCCTAATCTGAAAAAGCCTCTAATAATTCTTCAAG
oCF.38	GTTTATTTTAATAAAAGCATATATTAAAAATGGAGGGACTTAGAGTTGG
oCF.39	CCTCCATTTTTAATATATGCTTTTATTAAAATAAACTTCTTCGTGATCATG
oCF.40	CAAAATAATTCATGGGGGAGTGATCGTTAAATTTATACTGCAATCG

bioRxiv preprint doi: https://doi.org/10.1101/2024.07.29.605636; this version posted July 29, 2024. The copyright holder for this preprint (which was not certified by peer review) is the author/funder, who has granted bioRxiv a license to display the preprint in perpetuity. It is made available under aCC-BY 4.0 International license.

oCF.41	CGATCACTCCCCCATGAATTATTTTGTTTTTTTTATTTCAATTAC
oCF.42	GGAGGATGATTATTTTTGAAGTACCCTATACGACAAGTAGC
oCF.43	GTATAGGGTACTTCAAAAATAATCATCCTCCTAAGTACAAGCTTAATTG
oCF.70	TCTCACCATCACCATCACCATGGTTCTTCTATGACTATAGATAAGAAAAAATTCATTGAAAAATATGTAAAAGCTTTAGAAAGTAATACA
oCF.72	TCTCACCATCACCATCACCATGGTTCTTCTTTGAAGTACCCTATACGACAAGTAGC
oCF.78	GATCATGATGGTGATTATAAGGATCATGATATCGACTACAAAGACGATGACGACAAGTTGAAGTACCCTATACGACAAGTAGC
oCF.84	CAATTATCTACAGCTAAAAACGAAGATGGCACACCTGT
oCF.85	CTTCGTTTTTAGCTGTAGATAATTGTGCAGTTTCATCGATTTTGTATTCAATTAATTGAG
oCF.86	CATGGTGATGGTGATGGTGAGAAGAACCCATAAATAATCATCCTCCTAAGTACAAGCTTAATTG
oCF.87	GTCTTTGTAGTCGATATCATGATCCTTATAATCACCATCATGATCCTTATAATCCATAAATAATCATCCTCCTAAGTACAAGCTTAATTG
oCF.90	GATAATGTCCAGAAGGTCGATAGAAAGCGTGAGAAACAG
oCF.91	CTTTCTATCGACCTTCTGGACATTATCCTGTACAACATC
oCF.97	CTTGAATGTTCATAAATAATCATCCTCCTAAGTACAAGCTTAATTG
oCF.98	ACTTAGGAGGATGATTATTTATGAACATTCAAGAAGCAACTAAGATAGC
oCF.99	TAAACCACGTCCTATAATTGCTTCAATAATTCCTGGTCTCTAG
oCF.100	ATTATTGAAGCAATTATAGGACGTGGTTTAACCCGGGTAA
oCF.101	TCTCACCATCACCATCACCATGGTTCTTCTATGAACATTCAAGAAGCAACTAAGATAGC
oCF.102	AAGTCTATGATATTGCTATTTCTCATAGTACAAAAGATAAGAAGACAGTTG
oCF.103	TTTTGTACTATGAGAAATAGCAATATCATAGACTTTTTGAGATTGAATATTCTTATT
oCF.104	GGGTTAGTTTTCAAATAGAATACTTTGAAAATCTAAAAAAACCTATATATATAGTAGAGTCTCTTGAAGAATT
oCF.105	GATTTTCAAAGTATTCTATTTGAAAACTAACCCAATCAGATTGAACTGAA
oCF.106	GCGTAAAACAGCAATTTCATATAAATACTCTGAAGCAAAAGATTTAAGA
oCF.107	AGAGTATTTATATGAAATTGCTGTTTTACGCGCCAACTCTAAG
oCF.108	TGGATTGATTGGCAAATAGAATACTCAGTTAAACAAATGAAAAGAGG
oCF.109	CTGAGTATTCTATTTGCCAATCAATCCAATTACTTTCTTTCATATTAGG
oCF.110	TCTCACCATCACCATCACCATGGTTCTTCTTAAGAGTGATCGTTAAATTTATACTGCAATCG
oCF.111	CATGGTGATGGTGATGGTGAGAAGAACCTTTTCTTCTACAGATATTATAATTCGTTGCTTTTT
oCF.112	GGACTTAGAGTTGGCGCGTAAAACATTTATTTCATATAAATAC
oCF.113	TACGCGCCAACTCTAAGTCCCTCCATTTTTAATATATCTAATCTG

bioRxiv preprint doi: https://doi.org/10.1101/2024.07.29.605636; this version posted July 29, 2024. The copyright holder for this preprint (which was not certified by peer review) is the author/funder, who has granted bioRxiv a license to display the preprint in perpetuity. It is made available under aCC-BY 4.0 International license.

oCF.114	GTCTTTGTAGTCGATATCATGATCCTTATAATCACCATCATGATCCTT ATAATCTTTTCTTCTACAGATATTATAATTCGTTGCTTTTT
oCF.115	GATCATGATGGTGATTATAAGGATCATGATATCGACTACAAAGACGAT GACGACAAGTAAGAGTGATCGTTAAATTTATACTGCAATCG
oCF.116	TCTCACCATCACCATCACCATGGTTCTTCTTAAGAGTGATCGTTAAAT TTATACTGCAATCG
oCF.117	CATGGTGATGGTGATGGTGAGAAGAACCTTTTCTTCTACAGATATTAT AATTCGTTGCTTTTT
oCF.126	CAGGTGCTTACAAAGTAGGTGAAGGTCAAAAAATCGAAAC
OCF.127	CTTCACCTACTTTGTAAGCACCTGGTTTATCAGCC
oCF.136	TGTGAGCGGATAACAATTATATATTAAAAATGGAGGGACTTAGAGTTG G
oCF.137	CCATTTTTAATATATAATTGTTATCCGCTCACAATTCCAC
oCF.160	GTCTTTGTAGTCGATATCATGATCCTTATAATCACCATCATGATCCTT ATAATCCATGCTTTTATTAAAATAAACTTCTTCGTGATCATG

bioRxiv preprint doi: https://doi.org/10.1101/2024.07.29.605636; this version posted July 29, 2024. The copyright holder for this preprint (which was not certified by peer review) is the author/funder, who has granted bioRxiv a license to display the preprint in perpetuity. It is made available under aCC-BY 4.0 International license.

Supplementary Sequences 1. DNA sequences of *mhp* genes from escaper Φ80α-vir phages that avoid Sau-Thoeris immunity. The sequence of the codon for residue V273 is shown in red.

Escaper 1

ATGGAACAAACACAAAAATTAAAATTAAATTTGCAACATTTTGCGAGTAACAATGTTA
AACCGCAAGTATTTAACCCTGATAATGTAATGATGCACGAAAAGAAAGATGGCACG
TTGATGAATGAATTCACAACGCCCATCTTACAAGAGGTTATGGAAAACTCTAAAATT
ATGCAATTAGGTAAGTACGAACCAATGGAAGGTACTGAGAAGAAGTTTACTTTTTG
GGCTGATAAACCAGGTGCTTACTGGGTAGGTGAAGGTCAAAAAATCGAAACATCTA
AAGCTACATGGGTTAATGCTACTATGAGAGCGTTTAAATTAGGGGTTATCTTACCTG
TAACAAAAGAATTCTTGAATTATACTTATTCACAATTCTTTGAAGAAATGAAGCCTAT
GATTGCTGAAGCATTCTATAAAAAGTTTGATGAAGCGGGTATTTTGAATCAAGGTAA
CAATCCATTCGGTAAATCAATTGCGCAATCAATTGAAAAAACTAATAAGGTTATTAA
AGGTGACTTCACACAAGATAACATTATTGATTTAGAGGCATTACTTGAAGATGACGA
ATTAGAAGCAAATGCGTTTATCTCAAAAACACAAAACAGAAGCTTGTTACGTAAAAT
TGTAGATCCTGAAACGAAAGAACGTATTTATGACCGTAACAGTGATTCGTTAGACG
GTCTACCTGTGGTTAACCTTAAATCAAGCAACTTAAAACGTGGTGAATTAATCACTG
GTGACTTCGACAAATTGATTTATGGTATCCCTCAATTAATCGAATACAAAATCGATG
AAACTGCACAATTATCTACAGCTAAAAACGAAGATGGCACACCTGTAAACTTGTTTG
AACAAGACATGGTGGCATTACGTGCAACTATGCATGTAGCATTGCATATCGCTGAT
GATAAAGCGTTTGCTAAGTTAGTTCCTGCTGACAAAAGAACAGATTCAGTTCCAGG
AGAAGTTTAA

Escaper 2

ATGGAACAAACACAAAAATTAAAATTAAATTTGCAACATTTTGCGAGTAACAATGTTA
AACCGCAAGTATTTAACCCTGATAATGTAATGATGCACGAAAAGAAAGATGGCACG
TTGATGAATGAATTCACAACGCCCATCTTACAAGAGGTTATGGAAAACTCTAAAATT
ATGCAATTAGGTAAGTACGAACCAATGGAAGGTACTGAGAAGAAGTTTACTTTTTG
GGCTGATAAACCAGGTGCTTACTGGGTAGGTGAAGGTCAAAAAATCGAAACATCTA
AAGCTACATGGGTTAATGCTACTATGAGAGCGTTTAAATTAGGGGTTATCTTACCTG
TAACAAAAGAATTCTTGAATTATACTTATTCACAATTCTTTGAAGAAATGAAGCCTAT
GATTGCTGAAGCATTCTATAAAAAGTTTGATGAAGCGGGTATTTTGAATCAAGGTAA
CAATCCATTCGGTAAATCAATTGCGCAATCAATTGAAAAAACTAATAAGGTTATTAA
AGGTGACTTCACACAAGATAACATTATTGATTTAGAGGCATTACTTGAAGATGACGA
ATTAGAAGCAAATGCGTTTATCTCAAAAACACAAAACAGAAGCTTGTTACGTAAAAT
TGTAGATCCTGAAACGAAAGAACGTATTTATGACCGTAACAGTGATTCGTTAGACG
GTCTACCTGTGGTTAACCTTAAATCAAGCAACTTAAAACGTGGTGAATTAATCACTG
GTGACTTCGACAAATTGATTTATGGTATCCCTCAATTAATCGAATACAAAATCGATG
AAACTGCACAATTATCTACAGCTAAAAACGAAGATGGCACACCTGTAAACTTGTTTG
AACAAGACATGGTGGCATTACGTGCAACTATGCATGTAGCATTGCATATCGCTGAT
GATAAAGCGTTTGCTAAGTTAGTTCCTGCTGACAAAAGAACAGATTCAGTTCCAGG
AGAAGTTTAA

bioRxiv preprint doi: https://doi.org/10.1101/2024.07.29.605636; this version posted July 29, 2024. The copyright holder for this preprint (which was not certified by peer review) is the author/funder, who has granted bioRxiv a license to display the preprint in perpetuity. It is made available under aCC-BY 4.0 International license.

Escaper 3
ATGGAACAAACACAAAAATTAAAATTAAATTTGCAACATTTTGCGAGTAACAATGTTA
AACCGCAAGTATTTAACCCTGATAATGTAATGATGCACGAAAAGAAAGATGGCACG
TTGATGAATGAATTCACAACGCCCATCTTACAAGAGGTTATGGAAAACTCTAAAATT
ATGCAATTAGGTAAGTACGAACCAATGGAAGGTACTGAGAAGAAGTTTACTTTTTG
GGCTGATAAACCAGGTGCTTACTGGGTAGGTGAAGGTCAAAAAATCGAAACATCTA
AAGCTACATGGGTTAATGCTACTATGAGAGCGTTTAAATTAGGGGTTATCTTACCTG
TAACAAAAGAATTCTTGAATTATACTTATTCACAATTCTTTGAAGAAATGAAGCCTAT
GATTGCTGAAGCATTCTATAAAAAGTTTGATGAAGCGGGTATTTTGAATCAAGGTAA
CAATCCATTCGGTAAATCAATTGCGCAATCAATTGAAAAAACTAATAAGGTTATTAA
AGGTGACTTCACACAAGATAACATTATTGATTTAGAGGCATTACTTGAAGATGACGA
ATTAGAAGCAAATGCGTTTATCTCAAAAACACAAAACAGAAGCTTGTTACGTAAAAT
TGTAGATCCTGAAACGAAAGAACGTATTTATGACCGTAACAGTGATTCGTTAGACG
GTCTACCTGTGGTTAACCTTAAATCAAGCAACTTAAAACGTGGTGAATTAATCACTG
GTGACTTCGACAAATTGATTTATGGTATCCCTCAATTAATCGAATACAAAATCGATG
AAACTGCACAATTATCTACAGCTAAAAACGAAGATGGCACACCTGTAAACTTGTTTG
AACAAGACATGGTGGCATTACGTGCAACTATGCATGTAGCATTGCATATCGCTGAT
GATAAAGCGTTTGCTAAGTTAGTTCCTGCTGACAAAAGAACAGATTCAGTTCCAGG
AGAAGTTTAA

Escaper 4

ATGGAACAAACACAAAAATTAAAATTAAATTTGCAACATTTTGCGAGTAACAATGTTA
AACCGCAAGTATTTAACCCTGATAATGTAATGATGCACGAAAAGAAAGATGGCACG
TTGATGAATGAATTCACAACGCCCATCTTACAAGAGGTTATGGAAAACTCTAAAATT
ATGCAATTAGGTAAGTACGAACCAATGGAAGGTACTGAGAAGAAGTTTACTTTTTG
GGCTGATAAACCAGGTGCTTACTGGGTAGGTGAAGGTCAAAAAATCGAAACATCTA
AAGCTACATGGGTTAATGCTACTATGAGAGCGTTTAAATTAGGGGTTATCTTACCTG
TAACAAAAGAATTCTTGAATTATACTTATTCACAATTCTTTGAAGAAATGAAGCCTAT
GATTGCTGAAGCATTCTATAAAAAGTTTGATGAAGCGGGTATTTTGAATCAAGGTAA
CAATCCATTCGGTAAATCAATTGCGCAATCAATTGAAAAAACTAATAAGGTTATTAA
AGGTGACTTCACACAAGATAACATTATTGATTTAGAGGCATTACTTGAAGATGACGA
ATTAGAAGCAAATGCGTTTATCTCAAAAACACAAAACAGAAGCTTGTTACGTAAAAT
TGTAGATCCTGAAACGAAAGAACGTATTTATGACCGTAACAGTGATTCGTTAGACG
GTCTACCTGTGGTTAACCTTAAATCAAGCAACTTAAAACGTGGTGAATTAATCACTG
GTGACTTCGACAAATTGATTTATGGTATCCCTCAATTAATCGAATACAAAATCGATG
AAACTGCACAATTATCTACAGCTAAAAACGAAGATGGCACACCTGTAAACTTGTTTG
AACAAGACATGGTGGCATTACGTGCAACTATGCATGTAGCATTGCATATCGCTGAT
GATAAAGCGTTTGCTAAGTTAGTTCCTGCTGACAAAAGAACAGATTCAGTTCCAGG
AGAAGTTTAA

Wild-type

ATGGAACAAACACAAAAATTAAAATTAAATTTGCAACATTTTGCGAGTAACAATGTTA
AACCGCAAGTATTTAACCCTGATAATGTAATGATGCACGAAAAGAAAGATGGCACG
TTGATGAATGAATTCACAACGCCCATCTTACAAGAGGTTATGGAAAACTCTAAAATT

bioRxiv preprint doi: https://doi.org/10.1101/2024.07.29.605636; this version posted July 29, 2024. The copyright holder for this preprint (which was not certified by peer review) is the author/funder, who has granted bioRxiv a license to display the preprint in perpetuity. It is made available under aCC-BY 4.0 International license.

ATGCAATTAGGTAAGTACGAACCAATGGAAGGTACTGAGAAGAAGTTTACTTTTTG
GGCTGATAAACCAGGTGCTTACTGGGTAGGTGAAGGTCAAAAAATCGAAACATCTA
AAGCTACATGGGTTAATGCTACTATGAGAGCGTTTAAATTAGGGGTTATCTTACCTG
TAACAAAAGAATTCTTGAATTATACTTATTCACAATTCTTTGAAGAAATGAAGCCTAT
GATTGCTGAAGCATTCTATAAAAAGTTTGATGAAGCGGGTATTTTGAATCAAGGTAA
CAATCCATTCGGTAAATCAATTGCGCAATCAATTGAAAAAACTAATAAGGTTATTAA
AGGTGACTTCACACAAGATAACATTATTGATTTAGAGGCATTACTTGAAGATGACGA
ATTAGAAGCAAATGCGTTTATCTCAAAAACACAAAACAGAAGCTTGTTACGTAAAAT
TGTAGATCCTGAAACGAAAGAACGTATTTATGACCGTAACAGTGATTCGTTAGACG
GTCTACCTGTGGTTAACCTTAAATCAAGCAACTTAAAACGTGGTGAATTAATCACTG
GTGACTTCGACAAATTGATTTATGGTATCCCTCAATTAATCGAATACAAAATCGATG
AAACTGCACAATTATCTACAGTTAAAAACGAAGATGGCACACCTGTAAACTTGTTTG
AACAAGACATGGTGGCATTACGTGCAACTATGCATGTAGCATTGCATATCGCTGAT
GATAAAGCGTTTGCTAAGTTAGTTCCTGCTGACAAAAGAACAGATTCAGTTCCAGG
AGAAGTTTAA
